# Secular trends of cardiorespiratory fitness in children and adolescents over a 35-year period: Chronicle of a predicted foretold

**DOI:** 10.3389/fpubh.2022.1056484

**Published:** 2023-01-05

**Authors:** Mario Leone, Patrick Levesque, Sabrina Bourget-Gaudreault, Jean Lemoyne, Emilia Kalinova, Alain Steve Comtois, Hung Tien Bui, Luc Léger, Pierre Frémont, Maxime Allisse

**Affiliations:** ^1^Department of Health Sciences, Université du Québec à Chicoutimi, Chicoutimi, QC, Canada; ^2^Department of Medicine and Health Sciences, Université de Sherbrooke, Sherbrooke, QC, Canada; ^3^Department of Physical Activity Sciences, Université du Québec à Trois-Rivières, Trois-Rivières, QC, Canada; ^4^Department of Physical Activity Sciences, Université du Québec à Montréal, Montreal, QC, Canada; ^5^School of Kinesiology, Université de Montréal, Montréal, QC, Canada; ^6^Department of Kinesiology, Laval University, Quebec, QC, Canada; ^7^Department of Kinanthropology, Université de Sherbrooke, Sherbrooke, QC, Canada

**Keywords:** normative reference values, VO_2_max, functional capacity, secular trends, youth, cardiorespiratory fitness

## Abstract

**Background:**

In the context of concerns regarding the cardiorespiratory fitness (CRF) of youth populations, the aims of this study were: (1) to update reference values for the VO_2_max for school-aged Canadians and (2) to document secular trends in CRF after a 35-year interval.

**Methods:**

Between September 2014 and April 2017, the CRF of 3725 students (53.2% boys; 6.0 to 17.9 yrs) was determined using the 20-m shuttle run test. The sample was collected in 36 different schools from six cities of Québec (Canada).

**Results:**

Median values of VO_2_max decreased with age in both sexes (*p* ≤ 0.05). By the age of 10, more than 20% of boys showed VO_2_max values below the recommended value (42 ml·kg^−1^·min^−1^). At the age of 17, that proportion reached 56.8%. A similar proportion of 12 yrs girls (20%) were under the recommended minimal value (37 ml·kg^−1^·min^−1^) and that value reached 69.9% at the age of 17. Compared to 1982, the VO_2_max at age 17 has declined by 18% for boys and 12% for girls. The situation is worse in terms of functional capacity (number of stages completed) with an overall decrease of more than 30%.

**Conclusion:**

This study demonstrates that, compared to data obtained using the same methodology 35 years ago, the CRF and functional capacity of children and adolescents has declined to levels that should raise concerns from a public health perspective. Thus, the development of strategies to promote a physically active lifestyle in youth is more relevant than ever.

## Introduction

According to Health Canada (2016) (1) the prevalence of obesity in youth (5–17 years) has more than tripled over the last 30 years. One of the most common explanations is related to the marked decline in physical activity levels during childhood and adolescence (2, 3). In fact, some recent studies have also shown the huge impact of a physically active lifestyle on the prevention and management of multiple chronic health problems such as cardiometabolic risk factors, several cancers, mental health problems and more (4–6). Data from the Public Health Agency of Canada report (2016) (7) indicate that the vast majority of young Canadians fail to meet recommended levels of physical activity due to increased sedentary behaviors. In fact, nearly 91% of children and youth aged 5–17 do not reach the Canadian Physical Activity Guidelines (8) recommendation of 60 min of moderate to vigorous physical activity daily.

### Cardiorespiratory fitness

CRF is such a key determinant of health (4, 9) that it has been proposed as a vital sign that should be monitored in clinical practice (10). In childhood and adolescence specifically, poor CRF is a major precursor to the development of short-term and later-life cardiometabolic risk factors and chronic diseases (10–13). According to the WHO and based on several studies, VO_2_max which represents the maximal capacity of the organism to consume oxygen during maximum physical exertion, has long been considered the leading indicator of CRF (14–17). Although some authors have questioned the usefulness and relevance of field tests for the evaluation of the CRF (18, 19) there is a strong consensus in favor of the use of this type of test, particularly for population surveillance (20–22). In fact, over the last 4 decades, the most commonly used test to assess aerobic fitness in school is the 20-m shuttle run test (23). The popularity of this field test relies on the fact that it is easy to manage, requires little equipment and time, is inexpensive and can be administered to several individuals simultaneously. In 2019, Statistics Canada released a set of normative percentile reference values including CRF (24). For practical reasons, the aerobic test chosen was the Modified Canadian Aerobic Fitness Test (mCAFT), a submaximal step test used to estimate an individual's CRF. Due to the very different nature of the procedures, the two tests cannot be interchanged for population surveillance purposes since the estimated VO_2_max values will be different.

Thus, the first objective of this study was to provide an update of the reference values for VO_2_max for the Canadian youth population (aged 6–17). The second objective was document the suspected secular trends in youth CRF by comparing the data collected in 1982 by Léger et al. (23) with the results of the present study.

## Methods

### Design

This study is a descriptive comparative research with a cross-sectional design based on a large sample of children and adolescents from Québec (Canada).

### Participants

Between September 2014 and April 2017, a total of 3,725 students (boys = 1,983; girls = 1,742) were recruited for this study. The age varied between 6.0 and 17.9 years, which covers elementary and high school education in Canada. The participants were recruited in 36 different schools (elementary school = 24 and high school = 12) from six cities in the province of Québec (Montréal, Québec city, Saguenay, Trois-Rivières, Laval and Sherbrooke). The data was collected in the gymnasium of each school during physical education classes. Parents and students were informed of our presence and could indicate their refusal to participate in the project (a consent form was signed by the school authorities). The Institutional Ethical Committee Board (University of Québec in Chicoutimi) approved the project (no: 602-225-01).

### Selection of the school boards, schools and classrooms

A three-stage sampling approach was used for the selection of a representative number of school boards, schools and classrooms. Each school received an invitation letter in order to take part in the project. Following the sending of approximately 1,200 invitations to school principals, over 300 schools expressed their interest to participate in this project. Particular attention was also paid to the equitable representation of the various socioeconomic status in our sample through a socioeconomic school rating from the Québec government (Ministère de l'Éducation et de l'Enseignement Supérieur, 2017).

All schools and classrooms were randomly selected by lot. Apart from very rare exceptions, all students of the same class were assessed, thus eliminating a selection bias. If a chosen school would withdraw, a new draw was then carried out. All participants were free from illnesses, disabilities, or injuries that could have been aggravated by physical activity. The sample size required for conducting this study was determined from a Cohen's d power analysis in order to detect small effects (*d* < 0.1) with a 1-β = 0.95 for α = 0.05 using G Power software version 3.1.9.4. Thus, 1,564 youths per sex were required for a total of 3,128 participants.

### Anthropometric measures

Anthropometric variables were collected using procedures proposed by Lohman et al. (25). Body mass (BM) was noted to the nearest 0.1 kg using a Detecto scale (Missouri, USA). Body height (BH) was assessed using a Lafayette stadiometer (Louisiana, USA) at the nearest 0.1 cm. Body mass index (BMI) was also calculated. BMI (typical vs. overweight and obese youths) was classified according to age and sex as suggested by Cole et al. (26).

### Cardiorespiratory fitness

CRF was determined in accordance with the 20-m shuttle run test described and validated by Léger et al. (23). Briefly, the test took place in a standard size gymnasium of at least 25 m. The entire classroom (generally around twenty students) took up position on the starting line. Whenever a participant could no longer follow the required running speed, he or she was stopped and the number of the last completed stage was recorded. At the end of the test, the following information was then extracted or estimated for each student: the number of the final stage, the associated running speed (km·h^−1^) and the estimated VO_2_max value (ml·kg^−1^·min^−1^).

### Statistical analysis

All descriptive values are reported as mean ± standard deviation (SD). Confidence intervals (CI) were set at the 95% level. Cohen's effect sizes were calculated for various intergroup comparisons. The Shapiro-Wilk test for normality was compiled for each variable. When normality was violated, a Box-Cox transformation (27) was conducted using the following equation:


$$\begin{array}{r} BC=\left(VAR^{\text{L}}--1\right)\cdot L^{-1}whenL\neq 0\\ BC=Log\left(VAR\right)whenL=0 \end{array}$$

Where, BC, Box-Cox transformation; VAR, variable; L, lambda

The Box-Cox power exponential method, which smoothed the curves by cubic splines, has been used to create the curves.

Outliers were identified using the method proposed by Hoaglin et al. (28, 29). The equation reads as follows:


$$\begin{array}{r} \left[\left(Q75-Q25\right)\cdot g\right]-Q25\;for\;the\;lowest\;value\\ \left[\left(Q75-Q25\right)\cdot g\right]+Q75\;for\;the\;highest\;value \end{array}$$

Where Q75 = 3^rd^ quartile; Q25 = 4^th^ quartile; g = 2.2

Percentiles values were computed using the LMS method, (30) which read as follows:


$$\begin{array}{l} P=M\cdot {\left[1+LSZ\right]}^{1/\text{L}} \end{array}$$

Where, P = percentile; M = median; L = Lambda; S = coefficient of variation; S = Z-score for the desire percentile.

In order to be able to assess changes that have occurred between 1982 and 2017, the data from the present study were compared with the study carried out by Léger et al. (23) using an unpaired *T*-Test. Statistical analysis was produced by the IBM-SPSS software version 24.

## Results

Anthropometric (BM, BH and BMI) and cardiorespiratory (number of stages completed and VO_2_max) characteristics as a function of age and sex are shown in Table 1. From the age of 10, girls are heavier and taller than boys until about the age of 13 years, which is consistent with puberty in girls. The cardiorespiratory profile presents a different picture where boys already have higher values for all age groups. This difference is particularly marked for the functional component of the test, which is reflected by the number of stages completed in the 20-m shuttle run test.

**Table 1:** Anthropometric and cardiorespiratory profiles of boys and girls aged 6–17 years old.

	**Body mass**		**Height**		**BMI**		**VO**_**2**_ **max**		**Stages**	
	**(kg)**		**(cm)**		**(kg**·**m**^**−2**^**)**		**(ml**·**kg**^**−1**^·**min**^**−1**^**)**		**(number)**	

***Age***	**Mean**±**SD**	**CI (95%)**	**Mean**±**SD**	**CI (95%)**	**Mean**±**SD**	**CI (95%)**	**Mean**±**SD**	**CI (95%)**	**Mean**±**SD**	**CI (95%)**
**Boys**										
6.0–6.9 yrs	24.0 ± 5.1	22.7–25.2	121.3 ± 4.9	120.1–122.5	16.1 ± 3.2	15.3–16.8	48.6 ± 2.5	47.7–48.9	2.1 ± 1.0	1.9–2.4
7.0–7.9 yrs	25.2 ± 4.4	24.4–26.0	125.5 ± 5.5	124.5–126.4	15.9 ± 2.2	15.5–16.3	47.9 ± 2.6	47.5–48.4	2.6 ± 1.3	2.4–2.8
8.0–8.9 yrs	28.9 ± 5.6	28.1–29.8	132.1 ± 6.7	131.1–133.1	16.5 ± 2.9	16.1–16.9	47.5 ± 3.9	46.9–48.1	3.2 ± 1.7	3.0–3.5
9.0–9.9 yrs	31.4 ± 5.7	30.6–32.2	136.8 ± 6.9	135.7–137.8	16.8 ± 2.9	16.4–17.3	46.7 ± 4.5	46.0–47.3	3.6 ± 1.9	3.3–3.8
10.0–10.9 yrs	35.3 ± 7.6	34.3–36.4	142.1 ± 6.6	141.2–143.1	17.4 ± 3.2	16.9–17.8	45.9 ± 4.7	45.2–46.6	3.9 ± 2.0	3.6–4.2
11.0–11.9 yrs	40.1 ± 8.0	38.8–41.3	148.1 ± 7.2	146.9–149.2	18.3 ± 3.4	17.7–18.8	43.9 ± 4.5	43.2–44.6	3.8 ± 1.8	3.5–4.1
12.0–12.9 yrs	47.5 ± 12.2	45.8–49.1	153.6 ± 8.5	152.5–154.8	20.0 ± 4.4	19.4–20.6	44.8 ± 5.2	44.1–45.5	5.0 ± 2.0	4.7–5.2
13.0–13.9 yrs	54.4 ± 12.7	52.7–56.0	161.0 ± 8.8	159.9–162.2	20.9 ± 4.1	20.3–21.4	43.8 ± 4.9	43.1–44.4	5.3 ± 1.9	5.0–5.5
14.0–14.9 yrs	57.9 ± 10.4	56.3–59.5	166.4 ± 7.8	165.2–167.6	21.1 ± 4.1	20.5–21.7	44.6 ± 6.7	43.6–45.6	6.1 ± 2.5	5.8–6.5
15.0–15.9 yrs	63.5 ± 12.2	61.9–65.2	170.5 ± 7.0	169.6–171.5	21.9 ± 4.2	21.3–22.5	43.0 ± 6.9	42.1–43.9	6.1 ± 2.5	5.8–6.5
16.0–16.9 yrs	66.6 ± 11.8	64.7–68.4	172.8 ± 7.6	171.6–174.0	22.5 ± 5.0	21.7–23.3	42.8 ± 7.3	41.7–44.0	6.6 ± 2.5	6.2–7.0
17.0–17.9 yrs	71.0 ± 14.9	68.0–74.5	173.5 ±7.9	171.8–175.1	23.6 ± 4.8	22.6–24.6	40.3 ± 7.0	39.4–41.8	6.5 ± 2.4	6.0–7.0
**Girls**										
6.0–6.9 yrs	22.5 ± 3.7	21.6–23.5	120.1 ± 5.1	118.8–121.4	15.6 ± 1.9	15.1–16.0	47.5 ± 2.0	47.5–48.0	1.7 ± 0.8	1.5–1.9
7.0–7.9 yrs	25.0 ± 4.7	24.1–25.8	125.4 ± 5.5	124.4–126.3	15.8 ± 2.2	15.4–16.2	47.1 ± 2.5	46.6–47.5	2.3 ± 1.0	2.1–2.5
8.0–8.9 yrs	28.9 ± 7.7	27.6–30.2	130.8 ± 6.4	129.7–131.8	16.8 ± 3.8	16.2–17.4	46.3 ± 3.1	45.8–46.8	2.6 ± 1.3	2.6–2.8
9.0–9.9 yrs	31.0 ± 6.1	30.1–31.9	136.2 ± 6.6	135.2–137.1	16.7 ± 2.6	16.3–17.0	44.5 ± 2.6	44.1–44.9	2.6 ± 1.1	2.5–2.8
10.0–10.9 yrs	37.7 ± 8.9	36.3–39.1	144.1 ± 8.3	142.8–145.4	18.1 ± 3.4	17.5–18.6	43.8 ± 3.7	43.3–44.4	3.1 ± 1.5	2.9–3.3
11.0–11.9 yrs	42.3 ± 9.3	40.8–43.9	149.5 ± 7.7	148.3–150.8	18.8 ± 3.6	18.2–19.4	42.5 ± 3.3	42.0–43.1	3.3 ± 1.3	3.1–3.5
12.0–12.9 yrs	49.4 ± 11.8	47.8–50.9	154.7 ± 6.7	153.8–155.5	20.5 ± 4.4	20.0–21.1	41.6 ± 4.4	41.1–42.2	3.7 ± 1.7	3.5–4.0
13.0–13.9 yrs	52.1 ± 11.2	50.6–53.6	157.2 ± 6.9	156.3–158.2	21.1 ± 4.4	20.5–21.7	39.5 ± 4.6	38.9–40.1	3.7 ± 1.8	3.4–3.9
14.0–14.9 yrs	57.6 ± 13.2	55.5–59.8	157.9 ± 6.4	156.9–159.0	23.1 ± 5.1	22.2–23.9	37.3 ± 4.3	36.6–38.0	3.5 ± 1.6	3.2–3.7
15.0–15.9 yrs	57.4 ± 9.2	55.7–59.1	161.4 ± 7.6	160.0–162.8	22.0 ± 3.6	21.3–22.6	38.4 ± 5.6	37.4–39.4	4.6 ± 2.0	4.2–4.9
16.0–16.9 yrs	59.7 ± 10.8	57.9–61.6	162.3 ± 9.0	160.8–163.9	22.7 ± 4.4	22.0–23.5	35.2 ± 5.1	34.3–36.1	4.2 ± 1.9	3.9–4.5
17.0–17.9 yrs	60.9 ± 13.0	57.8–64.0	162.6 ± 8.8	160.4–164.7	22.7 ± 4.2	21.7–23.7	33.9 ± 5.5	32.6–35.2	4.0 ± 1.8	3.6–4.5

SD, Standard deviation; CI, Confidence interval; BMI, Body mass index.

**Figure 1:**
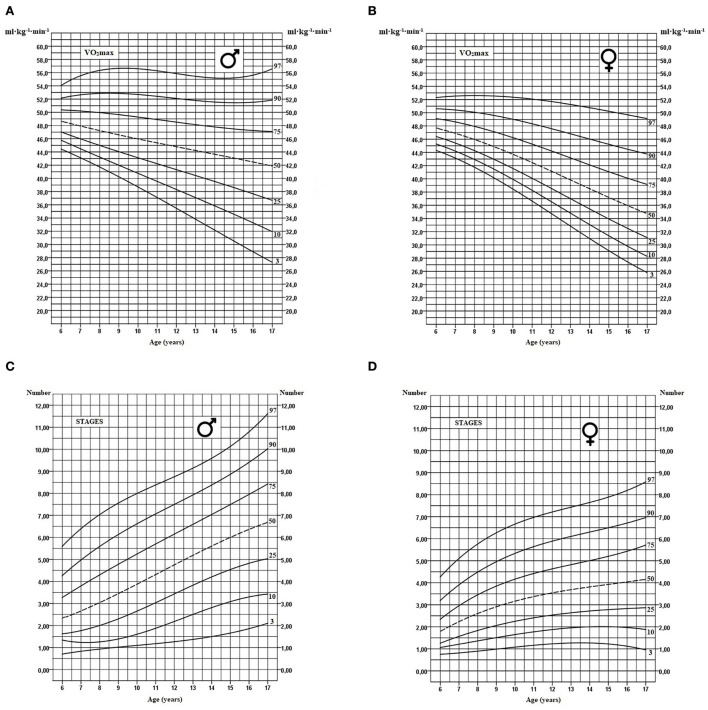
Age-specific smoothed percentile curves for VO_2_max and the number of stages completed estimated using the 20-m shuttle run test for boys **(A, C)** and girls respectively **(B, D)**.

Percentile curves of VO_2_max and functional capacity, for boys and girls aged 6 to 17, are presented in Figures 1A, B. Between age 6 and 17, the median values for VO_2_max declines by about 14% for boys and 27% for girls. Also, this trend appears to be strongly affected by the percentile (VO_2_max) reached in early childhood. For example, for the 25th percentile value, a decline of 21% for boys and 33% for girls is observed between the age of 6 and 17. In Figures 1C, D, the percentile curves of the number of stages during the 20-m shuttle run test provide useful information regarding the functional aspect of the cardiorespiratory capacity. Thus, individuals in the upper percentiles tend to considerably improve the number of stages completed throughout the physical growth period compared to individuals in the lower percentiles.

Tables 2, 3 provide the standardized values for VO_2_max and functional capacity (stages) respectively. All parameters included in the LMS method are reported for each year of chronological age (6–17 years) for both sexes. Additionally, values for the 3rd, 10th, 25th, 50th, 75th, 90th, and 97th percentiles are also shown.

**Table 2:** Percentile references values for VO_2_max according to age and gender in Québec children and adolescents (N = 3725).

	**Percentiles**										
	**N**	**L**	**M**	**S**	**3th**	**10th**	**25th**	**50th**	**75th**	**90th**	**97th**
**Boys**											
6.0–6.9 yrs	66	−1.55	48.3	0.052	44.1	45.3	46.7	48.3	50.1	51.8	53.7
7.0–7.9 yrs	123	−0.45	48.0	0.057	43.2	44.7	46.2	48.0	49.9	51.7	53.6
8.0–8.9 yrs	168	−1.70	47.5	0.083	41.4	43.1	45.0	47.5	50.4	53.4	56.9
9.0–9.9 yrs	178	−1.83	47.0	0.097	40.2	42.1	44.3	47.0	50.5	54.3	58.8
10.0–10.9 yrs	192	0.01	46.2	0.104	38.0	40.4	43.1	46.2	49.6	52.8	56.2
11.0–11.9 yrs	158	−0.70	43.9	0.104	36.5	38.6	41.0	43.9	47.2	50.5	54.2
12.0–12.9 yrs	213	0.43	44.6	0.115	35.5	38.3	41.2	44.6	48.1	51.4	54.8
13.0–13.9 yrs	232	1.06	43.8	0.110	34.7	37.6	39.8	43.8	47.0	50.0	52.8
14.0–14.9 yrs	180	0.54	44.8	0.150	32.0	36.6	40.4	44.8	49.4	53.8	58.3
15.0–15.9 yrs	216	1.09	43.1	0.160	30.0	34.2	38.4	43.1	47.7	51.8	55.9
16.0–16.9 yrs	162	0.90	42.8	0.171	29.3	33.5	37.9	42.8	47.8	52.3	56.8
17.0–17.9 yrs	95	0.90	41.6	0.181	27.8	32.1	36.6	41.6	46.7	51.3	55.9
**Girls**											
6.0–6.9 yrs	62	−1.00	47.5	0.042	44.0	45.1	46.2	47.5	48.9	50.2	51.6
7.0–7.9 yrs	119	−2.00	46.9	0.051	43.0	44.1	45.4	46.9	48.6	50.3	52.2
8.0–8.9 yrs	139	−1.90	46.4	0.067	41.5	42.9	44.4	46.4	48.6	51.0	53.6
9.0–9.9 yrs	180	−2.64	44.5	0.059	40.4	41.5	42.9	44.5	46.4	48.4	50.8
10.0–10.9 yrs	164	−1.05	43.7	0.089	37.7	39.4	41.3	43.7	46.4	49.0	52.0
11.0–11.9 yrs	143	−1.08	42.3	0.084	36.9	38.5	40.2	42.3	44.7	47.0	49.6
12.0–12.9 yrs	240	−0.96	41.9	0.105	35.0	36.9	39.1	41.9	45.1	48.4	52.2
13.0–13.9 yrs	218	−1.60	39.3	0.119	32.5	34.3	36.5	39.3	42.8	46.8	51.8
14.0–14.9 yrs	146	−0.70	37.1	0.115	30.3	32.2	34.4	37.1	40.2	43.4	47.0
15.0–15.9 yrs	123	0.30	37.9	0.145	28.4	31.2	34.3	37.9	41.8	45.5	49.4
16.0–16.9 yrs	135	−0.40	36.6	0.167	27.2	29.8	32.8	36.6	41.1	45.8	51.3
17.0–17.9 yrs	73	−0.70	34.4	0.162	26.1	28.4	31.0	34.4	38.1	43.0	48.4

N, Number of participants; L, lambda; M, median; S, coefficient of variation (SD·mean^−1^).

**Table 3:** Percentile reference values for the number of stages completed during the 20-m shuttle run test according to age and gender in Québec children and adolescents (N = 3725).

	**Percentiles**										
	**N**	**L**	**M**	**S**	**3th**	**10th**	**25th**	**50th**	**75th**	**90th**	**97th**
**Boys**											
6.0–6.9 yrs	66	0.25	2.22	0.519	0.75	1.00	1.50	2.25	3.00	4.00	5.25
7.0–7.9 yrs	124	0.34	2.71	0.487	1.00	1.25	2.00	2.75	3.75	4.75	6.00
8.0–8.9 yrs	168	0.23	3.35	0.514	1.00	1.50	2.25	3.25	4.75	6.25	8.00
9.0–9.9 yrs	178	0.34	3.85	0.531	1.25	1.75	2.75	3.75	5.50	7.00	9.00
10.0–10.9 yrs	192	0.63	4.19	0.501	1.00	1.75	2.75	4.25	5.75	7.25	8.50
11.0–11.9 yrs	158	0.63	3.67	0.480	1.00	1.75	2.50	3.75	5.00	6.25	7.50
12.0–12.9 yrs	213	0.69	5.13	0.405	1.75	2.75	3.75	5.00	6.50	8.00	9.50
13.0–13.9 yrs	232	1.00	5.50	0.351	1.75	3.00	4.25	5.50	6.75	8.00	9.25
14.0–14.9 yrs	180	0.66	6.18	0.397	2.25	3.25	4.50	6.25	8.00	9.50	11.25
15.0–15.9 yrs	217	1.00	6.00	0.400	1.50	3.00	4.50	6.00	7.50	9.00	10.50
16.0–16.9 yrs	162	0.86	6.57	0.378	2.25	3.50	5.00	6.50	8.25	9.75	11.50
17.0–17.9 yrs	95	0.81	6.59	0.372	2.25	3.50	5.00	6.50	8.25	9.75	11.50
**Girls**											
6.0–6.9 yrs	62	−0.60	1.73	0.432	0.75	1.00	1.25	1.75	2.50	3.50	5.25
7.0–7.9 yrs	120	0.01	2.25	0.462	1.00	1.25	1.50	2.25	3.00	4.00	5.25
8.0–8.9 yrs	139	0.12	2.84	0.492	1.00	1.50	2.00	2.75	4.00	5.25	6.75
9.0–9.9 yrs	180	0.26	2.67	0.440	1.00	1.50	2.00	2.75	3.50	4.50	5.75
10.0–10.9 yrs	165	0.34	3.20	0.498	1.00	1.50	2.25	3.25	4.50	5.75	7.25
11.0–11.9 yrs	143	0.38	3.16	0.389	1.25	1.75	2.25	3.25	4.00	5.00	6.00
12.0–12.9 yrs	240	0.48	3.74	0.449	1.25	2.00	2.75	3.75	5.00	6.25	7.50
13.0–13.9 yrs	218	0.31	3.81	0.484	1.25	2.00	2.75	3.75	5.25	6.75	8.50
14.0–14.9 yrs	146	0.62	3.15	0.465	1.00	1.50	2.25	3.25	4.25	5.25	6.50
15.0–15.9 yrs	123	0.63	4.16	0.429	1.25	2.00	3.00	4.25	5.50	6.75	8.00
16.0–16.9 yrs	133	0.37	4.30	0.472	1.50	2.25	3.00	4.25	5.75	7.50	9.25
17.0–17.9 yrs	73	0.61	4.18	0.460	1.25	2.00	3.00	4.25	5.50	7.00	8.50

N, Number of participants; L, lambda; M, median; S, coefficient of variation (SD·mean^−1^); The number of stages has been rounded to the nearest quarter. The speed at the first stage starts at 8.5 km·h^−1^ and than increase by 0.5 km·h^−1^ every minute.

**Figure 2:**
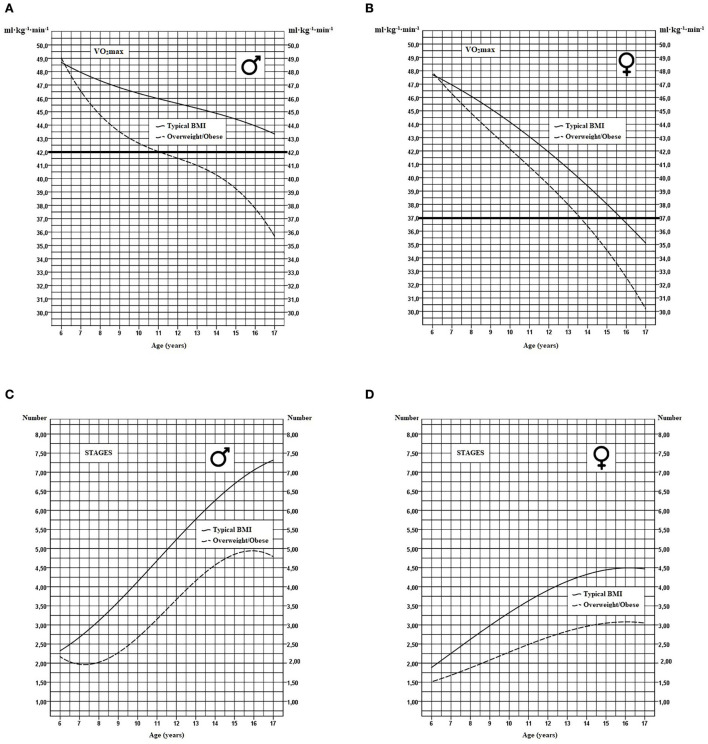
Modeling of VO_2_max curves and the number of stages completed in youth with typical BMI or with overweight/obese profile in boys **(A, C)** and girls **(B, D)**. The thick horizontal line represents the critical cutoff health zone.

The impact of BMI on VO_2_max was also examined (Figures 2A, B). As shown in Table 4, boys in the overweight/obese zone have VO_2_max values markedly lower than individuals with typical BMI across all age groups and this difference increases between the age of 14 and 17. In girls, a similar but less important difference is observed between overweight/obese individuals and those with typical BMI follows a slightly shifted curve which increases with age in favor of the former. For the functional aspect of the 20-m shuttle run test, a very large discrepancy is also observed when comparing individuals from the two BMI categories (Figures 2C, D).

**Table 4:** Comparison of VO_2_max and the number of stages completed in children and adolescents with typical or overweight/obese BMI profile.

	**Typical BMI**		**Overweight/Obese**		Δ **%**		**P-values**		**Cohen's d**	

	**Boys**	**Girls**	**Boys**	**Girls**	**Boys**	**Girls**	**Boys**	**Girls**	**Boys**	**Girls**
**6.0–6.9 yrs**										
VO_2_max	48.3 ± 2.6	47.3 ± 1.9	48.5 ± 2.6	48.3 ± 2.4	−0.4	−2.0	0.856	0.193	0.08	0.51
Stages	2.2 ± 1.2	1.7 ± 0.8	2.0 ± 1.0	1.5 ± 0.6	9.1	11.8	0.668	0.542	0.17	0.26
**7.0–7.9 yrs**										
VO_2_max	48.1 ± 2.8	47.3 ± 2.5	47.1 ± 1.9	45.7 ± 1.8	2.1	3.4	0.159	**0.007**	0.37	0.67
Stages	2.7 ± 1.3	2.3 ± 1.1	2.1 ± 1.0	1.6 ± 0.6	22.2	30.4	0.104	**0.000**	0.47	0.68
**8.0–8.9 yrs**										
VO_2_max	47.9 ± 3.9	46.7 ± 3.4	44.9 ± 3.3	44.6 ± 3.1	6.3	4.5	**0.000**	**0.001**	0.78	0.63
Stages	3.4 ± 1.7	2.9 ± 1.4	2.2 ± 1.2	1.8 ± 0.9	24.1	37.9	**0.000**	**0.000**	0.73	**0.84**
**9.0–9.9 yrs**										
VO_2_max	47.1 ± 4.5	44.7 ± 2.6	44.3 ± 4.0	43.4 ± 2.7	2.3	2.9	**0.003**	**0.013**	0.63	0.50
Stages	3.8 ± 1.9	2.7 ± 1.1	2.5 ± 1.6	2.2 ± 1.0	34.2	18.5	**0.001**	**0.014**	0.70	0.46
**10.0–10.9 yrs**										
VO_2_max	46.6 ± 4.6	44.2 ± 3.8	41.9 ± 3.4	42.7 ± 3.3	10.1	3.4	**0.000**	**0.031**	**1.06**	0.41
Stages	4.2 ± 2.0	3.3 ± 1.6	2.4 ± 1.3	2.5 ± 1.3	42.9	24.2	**0.000**	**0.007**	**0.94**	0.52
**11.0–11.9 yrs**										
VO_2_max	44.6 ± 4.5	43.1 ± 3.1	40.8 ± 3.6	40.1 ± 3.2	8.5	7.0	**0.000**	**0.000**	**0.87**	**0.96**
Stages	4.1 ± 1.9	3.5 ± 1.3	2.6 ± 1.3	2.4 ± 1.0	36.6	31.4	**0.000**	**0.000**	**0.83**	**0.88**
**12.0–12.9 yrs**										
VO_2_max	45.8 ± 5.0	41.6 ± 4.1	42.2 ± 4.7	41.8 ± 5.1	7.9	−0.5	**0.000**	0.714	0.73	0.05
Stages	5.3 ± 1.9	4.2 ± 1.7	4.0 ± 1.9	2.8 ± 1.3	24.5	33.3	**0.000**	**0.000**	0.68	**0.88**
**13.0–13.9 yrs**										
VO_2_max	44.7 ± 4.6	40.6 ± 4.5	41.4 ± 4.7	36.6 ± 4.1	8.1	9.9	**0.000**	**0.000**	0.71	**0.91**
Stages	5.6 ± 1.7	4.0 ± 1.8	4.4 ± 1.8	2.7 ± 1.5	21.4	32.5	**0.000**	**0.000**	0.69	0.75
**14.0–14.9 yrs**										
VO_2_max	46.0 ± 6.2	38.5 ± 4.2	39.8 ± 6.6	35.3 ± 3.4	13.5	8.3	**0.000**	**0.000**	**0.99**	**0.81**
Stages	6.7 ± 2.3	3.9 ± 1.6	4.4 ± 2.4	2.7 ± 1.3	34.2	30.8	**0.000**	**0.000**	**0.99**	**0.80**
VO_2_max	44.1 ± 6.7	39.6 ± 5.3	39.6 ± 6.8	34.2 ± 4.7	10.2	13.6	**0.000**	**0.000**	0.67	**1.05**
Stages	6.5 ± 2.4	5.0 ± 1.9	4.9 ± 2.4	3.1 ± 1.6	24.6	38.0	**0.000**	**0.000**	0.67	**1.04**
**16.0–16.9 yrs**										
VO_2_max	44.5 ± 6.8	36.4 ± 6.1	38.0 ± 5.9	34.3 ± 5.6	14.6	5.8	**0.000**	0.109	**0.99**	0.35
Stages	7.2 ± 2.4	4.5 ± 2.1	5.0 ± 2.0	3.6 ± 1.7	31.4	20.0	**0.000**	**0.040**	**0.96**	0.45
**17.0 yrs** +										
VO_2_max	42.9 ± 6.3	34.8 ± 4.8	35.4 ± 6.6	30.5 ± 6.3	17.5	12.4	**0.000**	**0.004**	**1.18**	**0.83**
Stages	7.2 ± 2.1	4.3 ± 1.7	4.7 ± 2.1	3.0 ± 2.0	34.7	30.2	**0.000**	**0.010**	**1.19**	0.73

Yrs, years; P-values in bold = significant at ≤ 0.05; Δ% = percentage of difference; Cohen's d = 0.20 marginal; 0.50 moderate; 0.80 large; Cohen d in bold = large effect (≥0.80).

**Figure 3:**
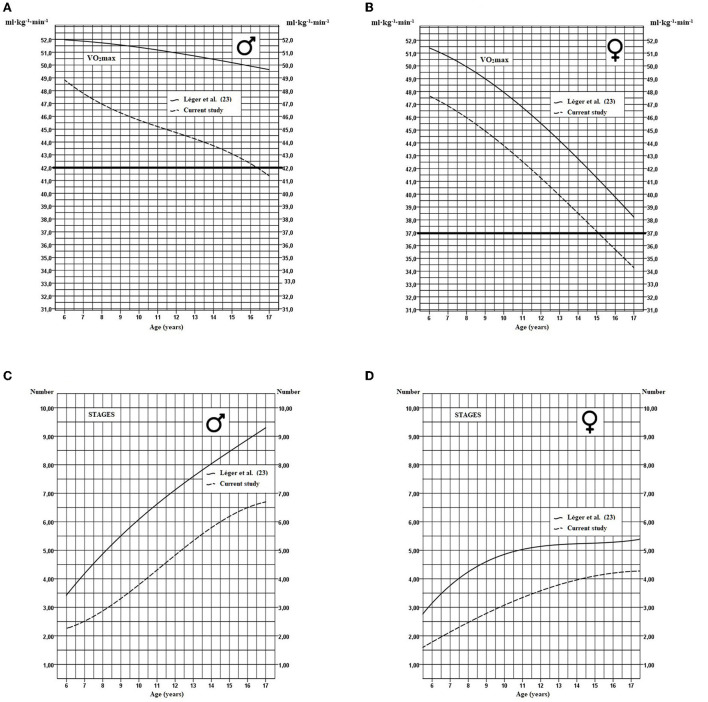
Modeling of the secular trend curves for median VO_2_max and for the number of stages completed between 1982 and 2017 for 6 to 17 yrs boys **(A, C)** and girls **(B, D)**. The thick horizontal line represents the critical cutoff health zone.

Secular trend for VO_2_max (Figures 3A, B) and the number of stages completed in the 20-m shuttle run test (Figures 3C, D) over a 35-year interval are illustrated by comparing data from the present study with data collected in 1982 using the same methodology (23). Over 35 years, median VO_2_max decreased by 7.6% for boys and 8.3% for girls at the age of 6 and this difference increases to nearly 18% in boys and 12.2% in girls by the age of 17 (Table 5). Compared to 1982, a significant decrease is also observed for all age groups for the number of stages completed for boys and girls.

**Table 5:** Comparison of VO_2_max and the number of stages completed in children and adolescents between 1982 and 2017.

	**Léger et al**. **(****23****)**		**Current study**		Δ **%**		**P-values**		**Cohen's d**	

	**Boys**	**Girls**	**Boys**	**Girls**	**Boys**	**Girls**	**Boys**	**Girls**	**Boys**	**Girls**
**6.0–6.9 yrs**										
VO_2_max	52.4 ± 2.8	51.8 ± 2.3	48.4 ± 2.5	47.5 ± 2.0	−7.6	−8.3	**0.000**	**0.000**	**1.43**	**1.96**
Stages	3.6 ± 1.4	3.4 ± 1.1	2.1 ± 1.1	1.7 ± 0.7	−41.7	−50.0	**0.000**	**0.000**	**1.15**	**1.74**
**7.0–7.9 yrs**										
VO_2_max	51.2 ± 3.3	50.3 ± 2.6	48.0 ± 2.7	47.0 ± 2.4	−6.3	−6.6	**0.000**	**0.000**	**1.02**	**1.3**
Stages	3.9 ± 1.6	3.5 ± 1.2	2.6 ± 1.3	2.2 ± 1.0	−33.3	−37.1	**0.000**	**0.000**	**0.86**	**1.13**
**8.0–8.9 yrs**										
VO_2_max	51.7 ± 3.9	49.8 ± 3.4	47.5 ± 3.9	46.3 ± 3.1	−8.1	−7.0	**0.000**	**0.000**	**1.11**	**1.06**
Stages	4.9 ± 1.8	4.1 ± 1.5	3.2 ± 1.7	2.7 ± 1.1	−34.7	−36.6	**0.000**	**0.000**	**0.96**	**1.04**
**9.0–9.9 yrs**										
VO_2_max	51.5 ± 4.4	49.2 ± 3.2	46.7 ± 4.5	44.5 ± 2.6	−9.3	−9.6	**0.000**	**0.000**	**1.10**	**1.57**
Stages	5.5 ± 1.9	4.5 ± 1.4	3.6 ± 1.9	2.7 ± 1.1	−41.8	−40.0	**0.000**	**0.000**	**1.26**	**1.38**
**10.0–10.9 yrs**										
VO_2_max	51.6 ± 4.2	46.8 ± 2.8	45.9 ± 4.8	43.8 ± 3.7	−11.2	−6.2	**0.000**	**0.000**	**1.33**	**0.92**
Stages	6.2 ± 1.8	4.9 ± 1.4	3.9 ± 2.0	3.1 ± 1.6	−37.1	−36.7	**0.000**	**0.000**	**1.23**	**1.26**
**11.0–11.9 yrs**										
VO_2_max	51.1 ± 4.5	47.5 ± 4.0	43.9 ± 4.6	42.6 ± 3.3	−14.1	−10.1	**0.000**	**0.000**	**1.60**	**1.24**
Stages	6.7 ± 1.8	5.2 ± 1.6	3.8 ± 1.8	3.3 ± 1.3	−43.3	−36.5	**0.000**	**0.000**	**1.61**	**1.25**
**12.0–12.9 yrs**										
VO_2_max	51.9 ± 5.2	46.7 ± 4.2	44.8 ± 5.2	41.6 ± 4.4	−13.7	−10.9	**0.000**	**0.000**	**1.37**	**1.19**
Stages	7.2 ± 2.0	5.5 ± 1.6	5.0 ± 2.0	3.7 ± 1.7	−30.6	−32.7	**0.000**	**0.000**	**1.10**	**1.09**
**13.0–13.9 yrs**										
VO_2_max	50.1 ± 5.2	44.4 ± 4.8	43.7 ± 4.8	39.5 ± 4.7	−12.6	−11.0	**0.000**	**0.000**	**1.23**	**1.04**
Stages	7.4 ± 2.0	5.3 ± 1.8	5.2 ± 1.8	3.7± 1.8	−29.7	−30.2	**0.000**	**0.000**	**1.15**	**0.89**
**14.0–14.9 yrs**										
VO_2_max	50.1 ± 5.2	41.7 ± 4.7	44.6 ± 6.7	37.3 ± 4.3	−11.0	−10.6	**0.000**	**0.000**	**0.94**	**0.97**
Stages	8.0 ± 1.9	4.8 ± 1.8	6.2 ± 2.5	3.5 ± 1.6	−23.8	−27.1	**0.000**	**0.000**	**0.88**	0.75
VO_2_max	50.2 ± 6.1	41.2 ± 5.1	43.1 ± 6.9	38.4 ± 5.6	−14.3	−6.8	**0.000**	**0.000**	**1.12**	0.55
Stages	8.5 ± 2.2	5.2 ± 1.8	6.1 ± 2.5	4.6 ± 2.0	−28.2	−11.5	**0.000**	**0.000**	**1.03**	0.32
**16.0–16.9 yrs**										
VO_2_max	50.0 ± 5.8	39.5 ± 5.0	42.8 ± 7.3	35.9 ± 6.0	−15.2	−9.1	**0.000**	**0.000**	**1.12**	0.68
Stages	8.9 ± 2.0	5.2 ± 1.7	6.6 ± 2.5	4.3 ± 2.0	−29.2	−17.3	**0.000**	**0.000**	**1.06**	0.67
**17.0 yrs** +										
VO_2_max	49.7 ± 5.9	38.6 ± 5.2	40.9 ± 7.4	33.9 ± 5.5	−17.7	−12.2	**0.000**	**0.000**	**1.38**	**0.89**
Stages	9.3 ± 2.0	5.5 ± 1.8	6.5 ± 2.4	4.1 ± 1.9	−30.1	−25.5	**0.000**	**0.000**	**1.27**	0.76

Yrs, years; P-values in bold = significant at ≤ 0.05; Δ% = percentage of difference (the negative sign indicates the decrease in percentage since 1982; Cohen's d = 0.20 marginal; 0.50 moderate; 0.80 large; Cohen d in bold = large effect (≥0.80).

Given that the difference in body mass between 1982 and 2017 could influence the VO_2_max observed between the two periods, BM normalization was carried out. The discrepancies observed between the 2 periods are not attenuated by BM standardization as shown in Table 6. Similarly, BM normalization did not affect the secular trends observed between 1982 and 2017 regarding the number of stages completed.

**Table 6:** Comparison of VO2max and the number of stages completed between Current study and Léger et al. (23) with adjustment for BM.

	**Boys**											
	**Current study**				**Léger et al**. **(****23****)**					**Statistics**		
**Age**	**N**	**Body mass**	**VO**_2_ **max**	**Stages**	**N**	**Body mass**	**P**	**N**	**VO**_2_ **max**	**P**	**Stages**	**P**
6	56	22.0 ± 2.8	48.3 ± 2.6	2.2 ± 1.1	89	23.4 ± 3.0	0.006	121	52.4 ± 2.8	**0**	3.6 ± 1.4	**0**
7	108	24.0 ± 3.2	48.1 ± 2.8	2.7 ± 1.3	221	25.2 ± 4.3	0.011	297	51.2 ± 3.3	**0**	3.9 ± 1.6	**0**
8	144	27.0 ± 3.7	47.9 ± 3.9	3.4 ± 1.6	211	28.0 ± 4.5	0.028	303	51.7 ± 3.9	**0**	4.9 ± 1.8	**0**
9	148	29.6 ± 4.4	47.1 ± 4.5	3.8 ± 1.9	200	31.8 ± 5.7	0	322	51.5 ± 4.4	**0**	5.5 ± 1.9	**0**
10	163	32.8 ± 5.1	46.6 ± 4.6	4.2 ± 1.9	253	34.6 ± 5.9	0.002	404	51.6 ± 4.2	**0**	6.2 ± 1.8	**0**
11	129	36.9 ± 5.5	44.6 ± 4.5	4.1 ± 1.8	247	38.8 ± 7.8	0.014	386	51.1 ± 4.5	**0**	6.7 ± 1.8	**0**
12	153	41.6 ± 7.4	45.8 ± 5.0	5.3 ± 1.9	206	42.7 ± 7.9	0.181	341	51.9 ± 5.2	**0**	7.2 ± 2.0	**0**
13	158	48.0 ± 8.3	44.7 ± 4.5	5.6 ± 1.7	233	47.8 ± 8.6	0.819	325	50.1 ± 5.2	**0**	7.4 ± 2.0	**0**
14	131	53.4 ± 7.0	46.0 ± 6.2	6.7 ± 2.2	237	53.4 ± 9.8	1	289	50.1 ± 5.2	**0**	8.0 ± 1.9	**0**
15	156	58.0 ± 7.7	44.1 ± 6.7	6.5 ± 2.4	254	58.3 ± 9.7	0.743	333	50.2 ± 6.1	**0**	8.5 ± 2.2	**0**
16	117	60.6 ± 8.0	44.5 ± 6.8	7.2 ± 2.3	245	62.6 ± 9.2	0.045	336	50.0 ± 5.8	**0**	8.9 ± 2.0	**0**
17	67	63.5 ± 6.6	42.9 ± 6.3	7.2 ± 2.1	161	64.5 ± 8.9	0.408	212	49.7 ± 5.9	**0**	9.3 ± 2.0	**0**
**Girls**												
6	51	21.8 ± 2.7	47.3 ± 1.9	1.7 ± 0.8	81	22.8 ± 2.9	0.05	112	51.8 ± 2.3	**0**	3.4 ± 1.1	**0**
7	97	23.3 ± 3.0	47.3 ± 2.5	2.3 ± 1.0	227	24.4 ± 3.6	0.009	299	50.3 ± 2.6	**0**	3.5 ± 1.2	**0**
8	111	26.2 ± 3.6	46.7 ± 3.1	2.9 ± 1.2	231	28.0 ± 5.2	0.001	308	49.8 ± 3.4	**0**	4.1 ± 1.5	**0**
9	152	28.9 ± 3.9	44.7 ± 2.6	2.7 ± 1.1	196	31.4 ± 5.6	0	322	49.2 ± 3.2	**0**	4.5 ± 1.4	**0**
10	128	34.2 ± 6.0	44.2 ± 3.8	3.3 ± 1.5	214	34.6 ± 7.0	0.59	335	46.8 ± 2.8	**0**	4.9 ± 1.5	**0**
11	118	39.5 ± 7.2	43.1 ± 3.1	3.5 ± 1.2	258	39.2 ± 8.5	0.74	382	47.5 ± 4.0	**0**	5.2 ± 1.6	**0**
12	165	43.4 ± 6.3	41.6 ± 4.1	4.2 ± 1.6	204	45.1 ± 9.0	0.041	292	46.7 ± 4.2	**0**	5.5 ± 1.6	**0**
13	161	47.2 ± 7.7	40.6 ± 4.5	4.0 ± 1.7	224	49.2 ± 9.0	0.023	298	44.4 ± 4.8	**0**	5.3 ± 1.8	**0**
14	93	49.7 ± 5.7	38.5 ± 4.2	3.9 ± 1.6	211	50.4 ± 7.3	0.412	260	41.7 ± 4.7	**0**	4.8 ± 1.8	**0**
15	89	53.5 ± 6.8	39.6 ± 5.3	5.0 ± 1.8	189	53.6 ± 7.1	0.912	260	41.2 ± 5.1	**0.012**	5.2 ± 1.8	0.366
16	102	56.0 ± 7.3	36.6 ± 5.9	4.5 ± 2.0	236	54.2 ± 7.8	0.048	332	39.5 ± 5.0	**0**	5.2 ± 1.7	**0.001**
17	54	56.0 ± 8.4	34.8 ± 4.8	4.4 ± 1.6	133	54.4 ± 7.4	0.199	155	38.6 ± 5.2	**0**	5.5 ± 1.8	**0**

N, number of participants; Body mass = kg; VO_2_max = ml·kg^−1^·min^−1^; Stages = number completed; P-values in bold = significant at ≤ 0.05.

## Discussion

This study provides recent reference values for the maximal aerobic 20-m shuttle run test in children and adolescents of the province of Québec (Canada). It also provides unique opportunity to directly compare recent CRF data with reference values initially documented for this test in the same geographic area and age group in 1982 (31).

### CRF reference values

According to different studies and regardless of age, it is estimated that a minimum VO_2_max value of approximately 42 ml·kg^−1^·min^−1^ in boys and 37 ml·kg^−1^·min^−1^ in girls is required to minimize the risk of developing severe health problems (32, 33). Considering these CRF thresholds, the reference values documented in this study raise a powerful red flag by showing that, by the age of 17 in boys and 15 in girls, the median VO_2_max value is below the minimal CRF level associated with favorable health outcomes later in life. These results are consistent with the reference values reported by Tomkinson et al. (6) in a metanalysis of 177 studies, most of which were published between 2000 and 2015.

Functional capacity is also affected, demonstrating that today's youth have a reduced ability to sustain moderate/intense effort. In fact, this decrease begins 1 year earlier than the decline in VO_2_max. This finding is certainly as worrying as the decrease in VO_2_max.

The results also indicate that the higher the CRF reached at a young age, the greater the chances that it will be maintained during the growth period. Assuming that this tendency persists later in life through adulthood, these results further support the notion that childhood CRF can contribute to prevent the development of cardiometabolic risk factors and diseases later in life (14).

### Effect of overweight/obesity on the CRF

Although obesity is recognized as a major cause of morbidity, (1, 32) a recent metanalysis indicated that it is not an independent factor of premature mortality (14). In the present study, data stratification for BMI (typical vs. overweight/obese) shows that, in the later group, a higher proportion evolves toward a VO_2_max value below the minimal CRF level associated with favorable health outcomes. In boys with the overweight/obese profile, it is noted that the critical median cutoff value of VO_2_max is crossed as soon as the age of 11, which never happens for the typical BMI group.

The number of stages completed as a function of age is also heavily impaired in the overweight/obese BMI group. These results further support the notion that lower CRF and functional capacity are factors that likely contribute to unfavorable cardiometabolic outcomes in youth with BMI that correspond to the definition of overweight and obesity. However, other factors also need to be taken into account since in girls with a normal BMI median VO_2_max will eventually fall below the recommended cutoff by the age of 16. Reduced physical activity combined with increased passive activities has been suggested as a likely factor that explains this situation (32, 34, 35).

### Secular trends in CRF

The great heterogeneity of CRF assessment procedures makes comparisons between studies complicated. In 1982, Léger and colleagues developed the 20-m shuttle run test and developed reference values for the CRF of youth living in Quebec (23). International reference values for the 20-m shuttle run test were recently developed by combining data from studies published up to 2015 (6). However, these more recent reference values do not allow comparison for specific population over time. The present study has the advantage of using the same test, administered under the same conditions, in the same cities and in the same school boards as 35 years earlier by Léger et al. (23).

This methodology resulted in a unique opportunity to objectively appreciate the evolution of CRF in youth between 1982 and 2017. The results confirmed an important decrease of CRF (estimated VO_2_max) and functional capacity (number of stages completed) in the study population. This difference tends to accentuate with age, with a VO_2_max decrease reaching nearly −18% for males and −12% for females at the age of 17. The functional impact of this situation is even more important in terms of the number of stages completed with an overall decrease of more than −30%. Furthermore, in 1982, all age groups of both sexes displayed VO_2_max values above the minimal recommended threshold associated with positive health outcomes. In the present study population, this is no longer the case from the age of 16 for boys and 15 for girls.

While some authors deny the fact that CRF has decreased over the last decades (18, 19) our results clearly show an alarming decline, both in relative (ml·kg^−1^·min^−1^) and functional values (number of stages). It has been suggested that the decrease in VO_2_max when expressed in relative values is predictable given the increase in body mass in youth over the past decades. However, data from the present study indicates that, after normalization for BM, significant differences remain for VO_2_max and for the number of stages completed in all age groups for both sexes. It may be noted that even when the body mass of Léger's cohort age groups was heavier, the relative and functional values remain markedly higher in their favor. Under these circumstances, it is reasonable to assume that body mass alone is insufficient to explain these differences.

It is assumed that, in addition to the body mass gain observed in recent decades in children and adolescents, increased time spent on sedentary activities is the factor that probably best explains the decrease in CRF (14, 21). Back in the 1980s when the 20-m shuttle run test was developed, computers and video games were in their infancy. With the development of the Internet and social networks youth became less physical active with an increase of time spent on sedentary activities (36, 37). As of 2016, combined data from 146 countries indicates that over 80% of adolescents do not meet the recommended levels of physical activity (38). Even more recently, the substantial reduction in physical activity due to containment measures related to COVID-19 is expected to further accelerate this decline (39).

### Reference values vs. standard values

In this paper, we use two distinct concepts that deserve to be explained. Based on the Centers for Disease Control and Prevention (DCC) in 2002, (40) reference values reflect the current situation without regard to its impact on health (what is). This information should not be interpreted as an objective to be achieved. This seems quite obvious as the VO_2_max values as well as the number of stages completed (i.e., functional capacity) have considerably decreased over the last decades. Thus, the role of the reference values is to make possible to measure the actual changes, and perhaps those that may occur in the future. They also allow comparison of current values with those from other studies.

On the other hand, the standard values represent what is minimally desirable in order to protect against certain potential health problems (what should be). In the case of VO_2_max, the minimum threshold should be around 42 ml in boys and 37 ml in girls. In order to stay above these thresholds, the reference values indicate that youths should follow at least around the 65th percentile curve throughout the growth period.

### Strengths and limitations

The large sample size (*n* > 3700) allows a valid representation of youths living in Québec (Canada). The test used to estimate the VO_2_max is internationally accepted as valid and reliable. The procedure used was repeated under the same conditions: same cities, same test and same school boards as the original 1982 study, which allows to assess the secular trends with a reduced number of biases. However, some limitations should also be noted. The cross-sectional nature of the data restricts inferences. VO_2_max values were estimated instead of measured directly, which affected the accuracy. Finally, although some towns were in suburban areas, cities in rural zones were not represented.

## Conclusion

While providing updated reference values for the 20-m shuttle run test, this study provides direct comparative evidence of an alarming decrease of CRF and functional capacity in a population of children and adolescents since the 1980s. This further highlights the threat of an epidemic of cardiometabolic pathologies in the near future. Thus, development of population surveillance tools and public health strategies to promote a physically active lifestyle is more important than ever.

## Data availability statement

The raw data supporting the conclusions of this article will be made available from the corresponding author, without undue reservation.

## Ethics statement

The studies involving human participants were reviewed and approved by the Université du Québec à Chicoutimi (CER). Written informed consent to participate in this study was provided by the participants' legal guardian/next of kin.

## Author contributions

ML was involved in the design and concept of the study, data collection, data analysis, drafted the initial, and final version of the manuscript. MA and EK co-ordinated, supervised data collection, involved in the study design, drafted the initial manuscript, reviewed, and revised the manuscript. AC, HB, and JL were involved in the study design, initial analyses, data collection, drafted the initial manuscript, reviewed, and revised the manuscript. LL and PF were involved in the data analysis, reviewed, and drafted the manuscript for important intellectual content. PL co-ordinated, supervised data collection, involved in initial data analysis, drafted the initial manuscript, reviewed, and revised the manuscript. SB-G was involved in the data collection, the initial data analysis, drafted the initial manuscript, reviewed, and revised the manuscript. All authors approved the final manuscript as submitted and agree to be accountable for all aspects of the work.
